# *BnVP1*, a novel vacuolar H^+^ pyrophosphatase gene from *Boehmeria nivea* confers cadmium tolerance in transgenic *Arabidopsis*

**DOI:** 10.1371/journal.pone.0308541

**Published:** 2024-08-19

**Authors:** Shoujing Zhu, Lei Chen, Zhonggui Zhang, Gang Chen, Nengbing Hu

**Affiliations:** 1 College of Agriculture, Anhui Science and Technology University, Fengyang, Anhui, China; 2 Anhui Sungu Agritech CO., LTD, Hefei, China; 3 Yichun University, Yichun, Jiangxi, China; National Taiwan University, TAIWAN

## Abstract

Plants have developed precise defense mechanisms against cadmium (Cd) stress, with vacuolar compartmentalization of Cd^2+^ being a crucial process in Cd detoxification. The transport of Cd into vacuoles by these cation / H^+^ antiporters is powered by the pH gradient created by proton pumps. In this study, the full-length cDNA of a vacuolar H^+^-pyrophosphatase (V-PPase) gene from *Boehmeria nivea* (ramie), *BnVP1*, was isolated using the rapid amplification of cDNA ends (RACE) method. The open reading frame (ORF) of *BnVP1* is 2292 bp, encoding a 763 amino acid V-PPase protein with 15 predicted transmembrane domains. Sequence alignment and phylogenetic analysis revealed that *BnVP1* belongs to the Type I V-PPase family. Quantitative RT-PCR assays demonstrated that *BnVP1* expression was significantly higher in ramie roots than in shoots. Cd treatments markedly induced *BnVP1* expression in both roots and leaves of ramie seedlings, with a more pronounced effect in roots. Additionally, *BnVP1* expression was significantly upregulated by the plant hormone methyl jasmonate (MeJA). Heterologous expression of *BnVP1* in transgenic *Arabidopsis* significantly enhanced V-PPase activity in the roots. The growth performance, root elongation, and total chlorophyll content of transgenic plants with high tonoplast H^+^-PPase (V-PPase) activity were superior to those of wild-type plants. Overexpression of *BnVP1* reduced membrane lipid peroxidation and ion leakage, and significantly increased Cd accumulation in the roots of transgenic *Arabidopsis* seedlings. This study provides new genetic resources for the phytoremediation of Cd-contaminated farmland.

## Introduction

Cadmium (Cd), often associated with lead (Pb), zinc (Zn), copper (Cu), mercury (Hg), and arsenic (As), is considered a non-essential and highly toxic heavy metal element to plants and animals, including humans. Cd can interfere with the antioxidant defense system, induce oxidative stress, and indirectly cause the production of reactive oxygen species (ROS), hydrogen peroxide (H_2_O_2_), and superoxide anion (O^2-^) when absorbed by plants [1]. The accumulation of ROS can lead to various negative effects, including membrane lipid peroxidation, ion leakage, protein denaturation, enzyme inactivation, and DNA damage [2]. Cd can also disrupt normal physiological activities in plants, such as inhibiting photosynthesis, decreasing chlorophyll content, altering water balance throughout the plant, interfering with the absorption of mineral nutrients, and even inducing plant death [3].

Plants have the ability to mitigate the negative effects of heavy metal ions on normal cellular physiological functions through mechanisms such as immobilization, efflux, and compartmentalization. The vacuole, being the largest organelle in plant cells, plays a critical role in these processes. The vacuole functions as a repository for ions and metabolites in cells primarily through H+ transporters on the tonoplast membrane. These include cation/H^+^ exchangers (CAXs), metal tolerance proteins (MTPs), magnesium proton exchangers (MHX), vacuolar iron uptake transporters (VIT), and Cu2+/H+ antiporters [4], which are involved in essential physiological processes such as the regulation of cell pH and the compartmentalization of salts and heavy metal ions [5]. There are two types of proton pumps on the tonoplast: vacuolar H+-ATPase (V-ATPase) and vacuolar H+-pyrophosphatase (V-PPase). Both are responsible for establishing the pH gradient between the vacuole and the cytoplasm. This pH gradient is utilized by the aforementioned proton-coupled antiporters to sequester cations like Cd2+ into the vacuole [6]. overexpression of V-PPase genes in *Cochliobolus sativus* [7], *Arabidopsis thaliana* [7], *Arabidopsis thaliana* [8], *Hordeum vulgare* [9] and *Nicotiana tobacum* [10, 11] has resulted in increased content of Cd, Cu, Zn, or nickel (Ni) in plants, demonstrating the crucial roles of V-PPase genes in heavy metal tolerance and accumulation.

*Boehemeria nivea* (ramie), also known globally as China grass, is a fiber crop of high economic value, widely distributed and cultivated in southern China. One of the primary products of ramie is textile fiber, which is renowned for its excellent moisture absorption, breathability, and antibiosis properties [12]. Previous studies have shown that ramie can not only grow normally in heavy metal mining areas but also accumulate varying concentrations of antimony (Sb), cadmium (Cd), mercury (Hg), and lead (Pb) [13, 14]. However, the molecular mechanisms by which ramie tolerates or accumulates Cd have not been thoroughly studied. In our previous research, a cDNA fragment (unigene 43810) from the root transcriptome of ramie under Cd stress, which shares high identities with V-PPase genes from other plant species, was found to be significantly induced by Cd. In this study, the rapid amplification of cDNA ends (RACE) method was used to isolate the full-length cDNA of the V-PPase gene (*BnVP1*). The coding sequence of *BnVP1* was then transformed into *Arabidopsis* seedlings for heterologous expression to investigate the possible roles of the V-PPase gene in ramie’s adaptation to Cd stress.

## Materials and methods

### Plant materials and growth conditions

The ramie cultivar "Zhongzhu 1" used in this study was planted at the agricultural experimental farm of Anhui Science and Technology University. For tissue-specific gene expression analysis, fresh samples of roots, stems, leaves, and flowers were collected from 2-year-old ramie plants grown in the field at the flowering stage. For gene expression pattern analysis under Cd stress, 10 cm long ramie shoots were cut and planted in plastic pots (20 cm diameter) filled with vermiculite. All pots were irrigated with half-strength Hoagland nutrient solution once a week until newly-rooted seedlings were established, which took 35 days. CdCl_2_·2.5H_2_O was then added to the solution to a final concentration of 50 μM, and the seedlings were irrigated once for Cd treatments. Root and leaf samples from seedlings treated with Cd for 0 h, 3 h, 6 h, 12 h, and 24 h were collected. For plant hormone treatments, the 35-day-old ramie seedlings were sprayed with 50 μM MeJA. After 0 h, 3 h, 6 h, 12 h, and 24 h of treatment, root and leaf samples were collected. All treatments were repeated three times independently. the fresh samples collected from the treatments were immediately frozen in liquid nitrogen and stored at -80 °C until their total RNA was extracted.

### Isolation of the full-length of BnVP1 gene and its promoter

To clone the full-length cDNA of the gene encoding *BnVP1*, a pair of specific primers, VP1-3FO and VP1-3FI (S1 Table), were designed for 3’ rapid amplification of cDNA ends (RACE) based on the Unigene 43810 identified from ramie transcriptome profiling under Cd exposure in our previous studies. [15]. The total RNA from the fresh leaves of 35-day-old ramie seedlings was extracted using the EasyPure Plant RNA Kit (TransGen, Beijing, China) following the manufacturer’s instructions. Then, 3’ RACE was performed using the 3’ RACE kit (BBI, Shanghai, China) according to the manufacturer’s protocols with the primers mentioned above. The purified RACE products were sequenced by Shenggong Biotechnological Ltd (Shanghai, China). Subsequently, the full-length *BnVP1* sequence was obtained by assembling the known fragment from unigene 43810 and the 3’ terminal sequence using DNAMAN 8.0 software. To verify the accuracy of the assembled sequence, a pair of specific primers, VP1-F and VP1-R (S1 Table), were designed to amplify the fragment containing the open reading frame (ORF) using the high-fidelity PCR enzyme PrimeSTAR (Takara, Beijing, China) according to the protocol, followed by sequencing. The Amplification program was: 98 °C for 10 s, 59 °C for 5 s, and 72 °C for 30 s, totaling 32 cycles. The cDNA sequence of *BnVP1* has been submitted to the National Center for Biotechnology Information (NCBI) Genbank database with an accession number of MW029619.

#### Isolation of the *BnVP1* gene promoter

Genomic DNA extracted from ramie cultivar ‘Zhongzhu No.1’ was used as the template to amplify the 1897 bp of genomic DNA sequence upstream from the *BnVP1* initiation codon using the primer pair VP1-PF and VP1-PR (S1 Table). The PCR amplification proceeded for 34 cycles of denaturation at 98°C for 10 s, primer annealing at 58°C for 15 s, and extension at 72°C for 30 s using PrimeSTAR Max DNA Polymerase (Takara, Beijing, China). The PCR product was examined by agarose gel electrophoresis, purified, and sequenced.

### Bioinformatic analysis

The ORF was predicted using ORF Finder in NCBI (https://www.ncbi.nlm.nih.gov/orffinder/). The physicochemical parameters of the protein were predicted using the ProParam tool on the EXPASY website (https://www.expasy.org/resources/protparam). Conserved domain prediction was performed with SMART (http://smart.embl-heidelberg.de) and CD Search in NCBI (https://www.ncbi.nlm.nih.gov/Structure/cdd/wrpsb.cgi). Prediction of subcellular localization was carried out using the WoLF PSORT II online tool (https://www.genscript.com/wolf-psort.html). The multiple alignment was performed using DNAMAN 8.0 software. A Phylogenetic tree was constructed using MEGA 5.0 software. The putative cis-regulatory elements in the promoter sequence were predicted with the PlantCARE database (http://bioinformatics.psb.ugent.be/webtools/plantcare/html/).

### qRT-PCR analysis

quantitative real-time PCR (qRT-PCR) was performed using the TransStart Top Green qPCR SuperMix (TransGen, Beijing, China) on an Applied Biosystems ViiA 7 real-Time PCR system according to the manufacturer’s instructions. For the expression analysis of *BnVP1* in different tissues of ramie, the total RNA from roots, stems, leaves, and flowers, as well as samples from different treatments of Cd or plant hormones, was reverse transcribed using the TransScript One-Step gDNA Removal and cDNA Synthesis SuperMix (TransGen Biotech, Beijing, China) following the manufacturer’s protocols. The actin gene of ramie (Accession NO. DQ665832.2) was used as a reference gene. The primers VP1-qF and VP1-qR used in the experiment are listed in S1 Table. Three independent biological replicates were used for each control and time point. Relative transcript levels were calculated using the comparative 2^–ΔΔCT^ method.

### Recombination plasmid construction and plant transformation

The full-length coding region of *BnVP1* was obtained by PCR using the specific primers VP1-BamHI-F and VP1-HindIII-R (S1 Table). the PCR product was then purified using the EZ-10 Spin Column DNA Gel Extraction Kit (Shenggong, Shanghai, China) and digested with BamHI and HindIII (Takara, Beijing, China). Subsequently, the restriction fragment was ligated into the pBI121 plasmid for expression from the strong CaMV 35S promoter, a plant binary expression vector, which had been digested with the same endonucleases, to generate the transformation vector pBI121-VP1. The recombinant plasmid was introduced into *Agrobacterium* tumefaciens (A. tumefaciens) strain EHA105 via electroporation and transformed into *Arabidopsis* Col-0 wild type by the *Agrobacterium*-mediated floral dip method [16]. The positively transformed seedlings were screened on 1/2 MS medium containing 30 mg··L^-1^ kanamycin, and then verified using PCR at the DNA level with the specific primers VP1-35S-F and VP1-SP-R. The homozygous lines of the T_3_ progeny were further identified by qPCR, which was performed as described above using the specific primers VP1-qF and VP1-qR. These primers were also used for the following Cd treatments. The primers used for PCR amplification are provided in S1 Table.

### Determination of tonoplast H^+^-PPase

WT and transgenic *Arabidopsis* seedlings, aged 28 days, were cultivated in plastic pots (6 cm diameter) filled with nutrient-rich soil within a greenhouse environment set at 22 °C, with a photoperiod of 16 hours light and 8 hours dark. These seedlings received weekly irrigation with an equal amount of half-strength Hoagland nutrient solution [17]. After this, root samples were collected for subsequent analysis of tonoplast H^+^-PPase (V-PPase). The isolation of vesicles and enzyme assays followed established protocols [18, 19], involving the collection of Tonoplast-enriched membrane vesicles through sucrose density gradient ultracentrifugation. The activity of V-PPase was determined by quantifying the release of inorganic phosphate (Pi).

### Cd stress assays

#### Phenotypic analysis

To observe phenotypic responses to Cd stress, seeds from both wild-type (WT) and the T3 generation of transgenic *Arabidopsis* underwent sterilization with 75% alcohol for 1 minute followed by 10% NaClO for 10 minutes, and were subsequently rinsed three times with sterilized distilled water. These seeds were then plated on 1/2 Murashige and Skoog (MS) medium containing either 0 or 50 μM Cd. After subjecting the plates to 2 days of cold treatment at 4 °C, they were transferred to growth chambers set at 22 °C with a 16-hour-light/8-hour-dark photoperiod for 4 days. Measurements of seedling growth and root elongation for both WT and transgenic lines were conducted from the 5th to the 10th day after treatment. Additionally, fresh leaves were collected on the 10th day for chlorophyll determination, following established protocols [20]. Briefly, 0.1 g of fresh leaves from 10-day-old *Arabidopsis* plants were ground in 95% alcohol solvent and then incubated at 4 °C in the dark for 24 hours before centrifugation at 5000 rpm for 10 minutes. the absorbance of the extracted chlorophyll a and b was measured spectrophotometrically at 649 nm and 665 nm using a Shimadzu UV-1800 instrument (Kyoto, Japan). total chlorophyll content was calculated based on previously described methods [21], specifically:

$$Total\;chlorophyll\;content\;\left(mg\cdot g^{-1}\;FW\right)=\frac{18.08\times A_{649}+6.63\times A_{665}}{fresh\;weight}$$

#### Determination of ion leakage and MDA

To determine ion leakage and malondialdehyde (MDA) content, 14-day-old *Arabidopsis* seedlings cultivated in 6 cm diameter plastic pots filled with vermiculite within a greenhouse at 22 °C with a 16-hour-light/8-hour-dark photoperiod were irrigated once with 50 ml of half-strength Hoagland nutrient solution [17] containing either 0 or 50 μM Cd, and allowed to grow continuously for 7 days. Subsequently, fresh roots of *Arabidopsis* seedlings were collected for ion leakage and MDA content determination. The relative ion leakage of roots was assessed following established procedures [22]. Briefly, fresh roots were delicately rinsed with distilled water to remove electrolytes, then cut into 5 mm long pieces. These samples were then placed in 50 ml glass vials filled with 20 ml of distilled water and subjected to vacuum infiltration for 10 minutes. Following this, the samples were incubated in the dark overnight at room temperature. The initial electrical conductivity (EC1) was measured using a conductivity meter (INESA, China). Subsequently, the root samples were boiled for 10 minutes, and the total electrical conductivity (EC2) was measured after the vials cooled to room temperature. The relative ion leakage was calculated using the following formula:

$$Ion\;leakage\;(\%)=\frac{EC1}{EC2}\times 100$$

MDA was extracted from the roots using 5% trichloroacetic acid (TCA) and measured according to the method described in our previous study [23]. MDA content is expressed as nmol·L^-1^.

### Determination of Cd content

The wild-type (WT) and *BnVP1* transgenic plants were cultivated in plastic pots with a diameter of 6 cm, filled with vermiculite. They were irrigated weekly with half-strength Hoagland’s nutrient solution for a duration of 4 weeks. Subsequently, the seedlings were subjected to watering with nutrient solution containing 50 μM Cd and allowed to grow continuously for one week. Following this growth period, the samples were harvested, washed with tap water, and rinsed three times with deionized water. the roots and shoots were then separated and dried until reaching a constant weight. The concentration of Cd in the root and shoot samples was determined using flame atomic absorption spectroscopy (AAS) with HNO_3_-HClO_4_ digestion, following the methods previously described by Jedrzejczak et al [24]. There were three experimental replicates.

### Statistical analysis

All experiments described in the study were conducted in triplicate across three independent biological trials. Data analysis was performed using Microsoft Excel 2021 software (Microsoft Corp., Redmond, WA, USA) and IBM SPSS Statistics 17 software (SPSS Inc., Chicago, IL, USA). Paired t-tests were utilized to evaluate the significance of differences between groups. Data were presented as mean ± standard error of the mean (SEM). Graphs was generated using the Origin 8.5 software (Microcal Software, Northampton, MA, USA).

## Results

### Isolation and sequence analysis of *BnVP1*

**Fig 1:**
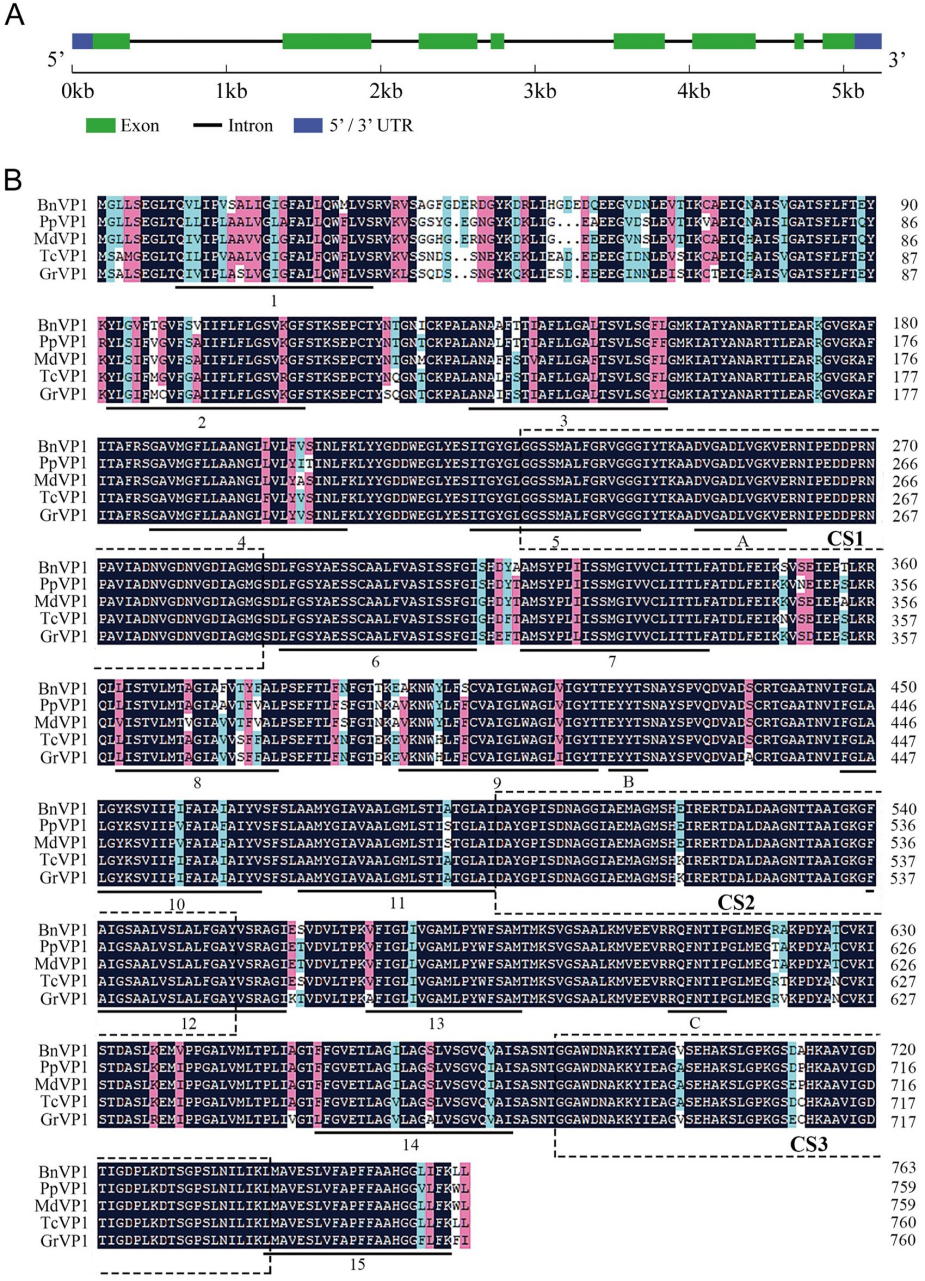
Gene structure and conserved domain analyses of *BnVP1*. **(A)** Exon/intron structure analysis of *BnVP1* gene. Green boxes, black lines and blue boxes represent the exons, introns and 5’ or 3’ untranslated regions (UTR), respectively. **(B)** Multiple alignment of the inferred amino acid sequences of BnVP1 and V-PPase proteins across plant species. The shaded sequences highlight conserved amino acid residues, with pink and light green indicating similar residues, and black showing fully conserved residues. The black underlined sequences denote transmembrane regions. The three conserved domains, CS1, CS2, and CS3, are indicated by dotted boxes. Additionally, the three conserved motifs are denoted by the letters A, B, and C. GenBank accession numbers of the proteins are as follows: PpVP1 (XP_007208066.1) from *Prunus persica*, MdVP1 (XP_008359855.1) from *Malus domestica*, TcVP1 (XP_017977384.1) from *Theobroma cacao*, GrVP1 (XP_012476518.1) from *Gossypium raimondii*.

In our previous study, we analyzed the transcriptome of the ramie cultivar "Zhongzhu 1" under cadmium (Cd) stress [15]. We identified an unigene, designated as 43810 (lacking the 3’-terminal), which was significantly induced by Cd treatments. This unigene exhibited a high level of sequence identity with vacuolar H+-pyrophosphatase (V-PPase) genes from other plants. Subsequently, the full-length cDNA of the V-PPase gene, with a length of 2600 bp, was isolated from "Zhongzhu 1" using RACE-PCR and RT-PCR methods (S1 Fig), and was designated as *BnVP1* (GenBank Accession No. MW029619). Gene structure analysis revealed that the full-length DNA of *BnVP1* gene is 5247 bp, containing eight exons, seven introns, a 5’ untranslated region (5’ UTR) of 133 bp, and a 3’ untranslated region (3’ UTR) of 175 bp (Fig 1A). The length of open reading frame (ORF) is 2292 bp, which encodes a putative vacuolar V-PPase consisting of 763 amino acids in length (Fig 1B, S2 Fig). The physicochemical properties analysis of *BnVP1* revealed several key characteristics. this protein is predicted to have a molecular mass of 80 kDa and an isoelectric point (pI) of 5.05. Analysis of the secondary structure using the server NPS@ indicated that BnVP1 predominantly consists of α-helices (55.57%), followed by random coils (33.29%) and extended strands (11.14%). Furthermore, transmembrane region analysis conducted using TMHMM v2.0 suggested that BnVP1 is a transmembrane protein, with fifteen putative transmembrane domains (Fig 1). Conserved domain analysis revealed that BnVP1 belongs to the H+-translocating inorganic pyrophosphatase family (Accession No. PLN02255, e-value: 0e-00). This classification is supported by the presence of three typical conserved domains: CS1 (amino acid position 230 to 289), CS2 (amino acid position 497 to 556), and CS3 (amino acid position 684 to 740), as observed in the topological model of H+-ppase [25]. The conserved motif D250VGADLVGK[VI]E, crucial for binding and catalytic functions, was found within the CS1 domain. Moreover, two other conserved sequences (E_420_YYTS and R_607_QFNTIP), which belong to the type I V-PPase family, were also identified within the BnVP1 amino acid sequence (Fig 1). Subcellular localization prediction showed that BnVP1 is an integral membrane protein localized in the vacuolar membrane.

**Fig 2:**
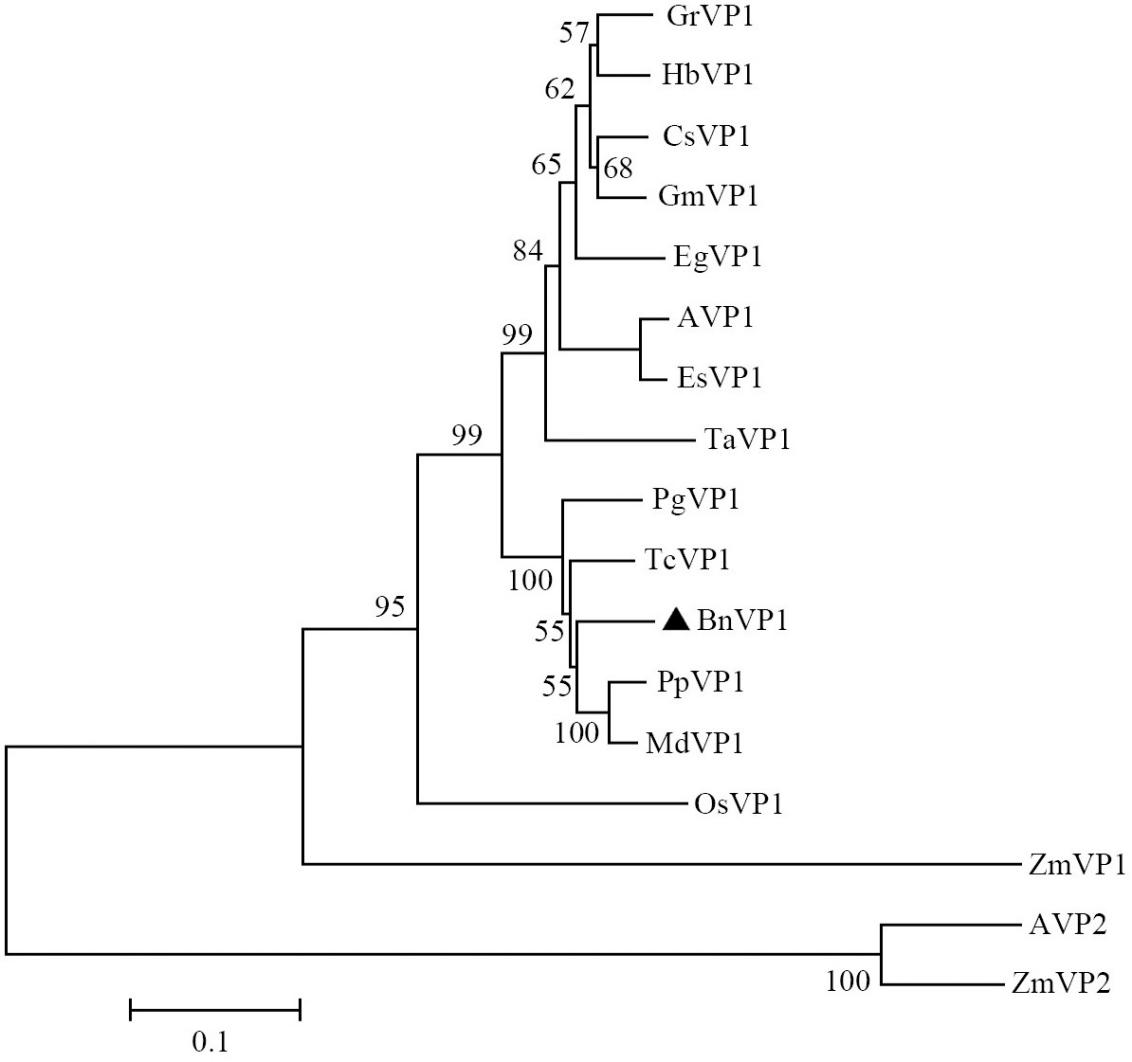
Phylogenetic tree of V-PPase from ramie and other 16 plant species. The scale bar represents 0.1 amino acid substitutions per site. The numbers displayed in the interior branches refer to the bootstrap values (%) obtained after 1000 replications. GenBank accession numbers of the proteins are as follows: GrVP1 (XP_012443156.1) from *Gossypium raimondii*, HbVP1 (AAS66771.1) from *Hevea brasiliensis*, CsVP1 (XP_028109862.1) from *Camellia sinensis*, GmVP1 (XP_003531725.1) from *Glycine max*, EgVP1 (XP_010927355.1) from *Elaeis guineensis*, AVP1 (P31414.1) from *Arabidopsis thaliana*, EsVP1 (AAR08913.2) from *Eutrema salsugineum*, TaVP1 (AAP55210.1) from *Triticum aestivum*, PgVP1 (XP_031404809.1) from *Punica granatum*, TcVP1 (XP_017977384.1) from *Theobroma cacao*, PpVP1 (XP_007208066.1) from *Prunus persica*, MdVP1 (XP_008359855.1) from *Malus domestica*, OsVP1 (XP_015637888.1) from *Oryza sativa*, ZmVP1 (ONM04875.1) from *Zea mays*, AVP2 (AAF31163.1) from *Arabidopsis thaliana*, ZmVP2 (ABK51382.1) from *Zea mays*.

The BLASTP similarity search conducted against the NCBI database revealed that BnVP1 exhibits high sequence similarity with type I V-PPases from various plant species, including *Prunus persica* (XP_007208066.1; 91.87%), *Malus domestica* (XP_008359855.1; 91.61%), *Theobroma cacao* (XP_017977384.1; 92.27%) and *Gossypium raimondii* (XP_012476518.1; 90.04%) (Fig 1). To analyze the evolutionary relationships among V-PPase proteins, a phylogenetic tree was constructed using the neighbor-joining method implemented in MEGA 5.0 software. This analysis included BnVP1 and sixteen other plant V-PPase proteins. The resulting phylogenetic tree revealed two distinct clades: VP1 and VP2 proteins. BnVP1 was found to be closely related to twelve previously reported type I V-PPases, including AVP1 from *Arabidopsis*. However, AVP2 was observed to be nested within a separate lineage, alongside an ortholog from *Zea mays* (ZmVP2), forming a distinct branch separate from the lineage containing BnVP1 and AVP1 (Fig 2).

#### Analysis of cis-acting elements in BnVP1 promoter

A 2000 bp DNA fragment containing the *BnVP1* promoter was isolated using the specific primers VP1-PF and VP1-PR (S1 Fig). The putative cis-acting elements within the promoter were predicted using the PlantCARE server. The results indicated that the *BnVP1* promoter contains the basic core elements, such as CAAT-box and TATA-box, but also abiotic stress and plant hormone responsive elements, such as the antioxidant response element (ARE), the low temperature response element (LTR), the MYB binding site that is involved in drought (MBS), the MeJA responsive element (CGTCA-motif), and the auxin responsive element (AuxRR-core) (Table 1).

**Table 1:** A sample of the predicted cis-acting elements identified in the promoter region of *BnVP1*.

Name	Amount	Signal Sequence	Function
ARE	5	AAACCA	cis-acting regulatory element essential for the anaerobic induction
AuxRR-core	1	GGTCCAT	cis-acting regulatory element involved in auxin responsiveness
Box 4	4	ATTAAT	part of a conserved DNA module involved in light responsiveness
CAAT-box	51	CAAT, CAAAT, CCAAT,	common cis-acting element in promoter and enhancer regions
TATA-box	39	TATA, TATAA, TATAAA, ATATAA, TATATA,	core promoter element around -30 of transcription start
CAT-box	1	GCCACT	cis-acting regulatory element related to meristem expression
CGTCA-motif	1	CGTCA	cis-acting regulatory element involved in the MeJA-responsiveness
GATA-motif	2	AAGGATAAGG, AAGATAAGATT	part of a light responsive element
GT1-motif	5	GGTTAA, GGTTAAT	light responsive element
I-box	2	AAGATAAGGCT, AGATAAGG	part of a light responsive element
LAMP-element	2	CTTTATCA	part of a light responsive element
LTR	1	CCGAAA	cis-acting element involved in low-temperature responsiveness
MBS	2	CAACTG	MYB binding site involved in drought-inducibility

### Analysis of the expression patterns of the *BnVP1* gene

**Fig 3:**
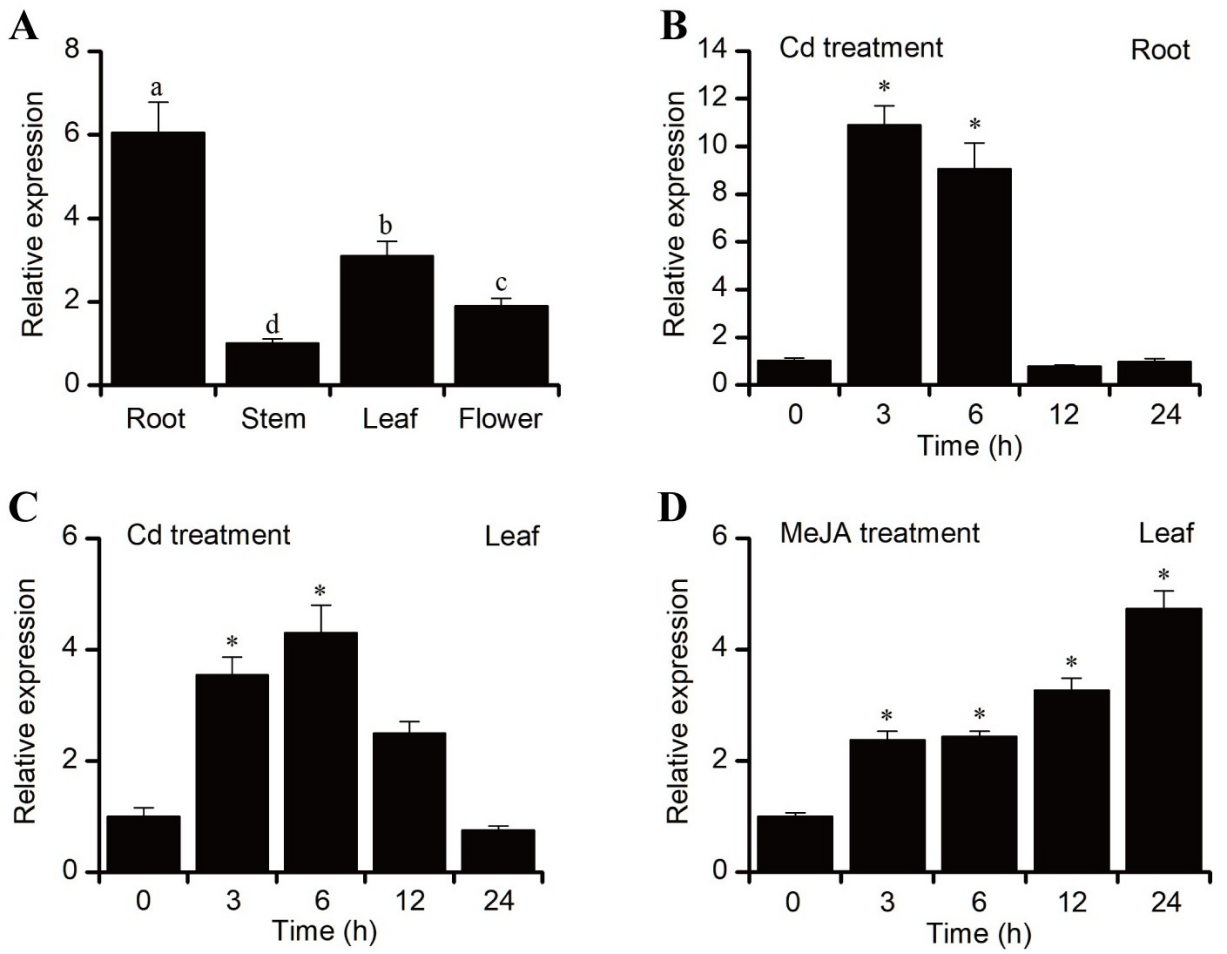
Expression patterns of *BnVP1* in ramie seedlings. **(A)** Transcript levels of *BnVP1* in different tissues of 35-day-old ramie seedlings. **(B, C)** *BnVP1* expression in the root or leaf of ramie seedlings following exposure to 50 μM Cd for 0, 3, 6, 12, 24 h. **(D)** *BnVP1* expression levels in the leaves of ramie seedlings treated with 50 μM MeJA for 0, 3, 6, 12, 24 h. Data are shown as the mean ± SE of three biological replicates. The asterisk represents a statistically significant difference when compared with untreated controls (*P*<0.05).

For the tissue-specific expression analysis, the expression patterns of the *BnVP1* gene in various tissues of 2-year-old ramie plants were examined using qRT-PCR. The results demonstrated that *BnVP1* was expressed in the root, stem, leaf, and flower of ramie. Notably, the highest expression level of *BnVP1* was observed in the root, followed by the leaf, with the lowest expression in the stem (Fig 3A). To investigate the response of *BnVP1* to Cd stress, 35-day-old ramie seedlings were treated with 50 μM Cd for 0, 3, 6, 12, and 24 hours, after which the expression patterns of *BnVP1* mRNA were assessed by qRT-PCR. The findings revealed that the transcript level of BnVP1 increased rapidly in both roots and leaves after 3 hours of Cd treatment. The highest transcript levels of *BnVP1* in the roots and leaves were 10.9- and 4.3-fold higher than the control, respectively. However, these levels declined to near-control levels after 12 and 24 hours, respectively (Fig 3B and 3C). Given the predicted cis-elements in the BnVP1 promoter, we also investigated the expression levels of BnVP1 under MeJA treatment. The results indicated that the transcript level of BnVP1 was significantly induced by MeJA, reaching its peak at 24 hours, with a 4.7-fold increase compared to the control (Fig 3D). These findings suggest that the expression of BnVP1 can be induced by both Cd stress and the plant hormone MeJA.

### Ectopic expression of *BnVP1* improves V-PPase activity in transgenic lines

**Fig 4:**
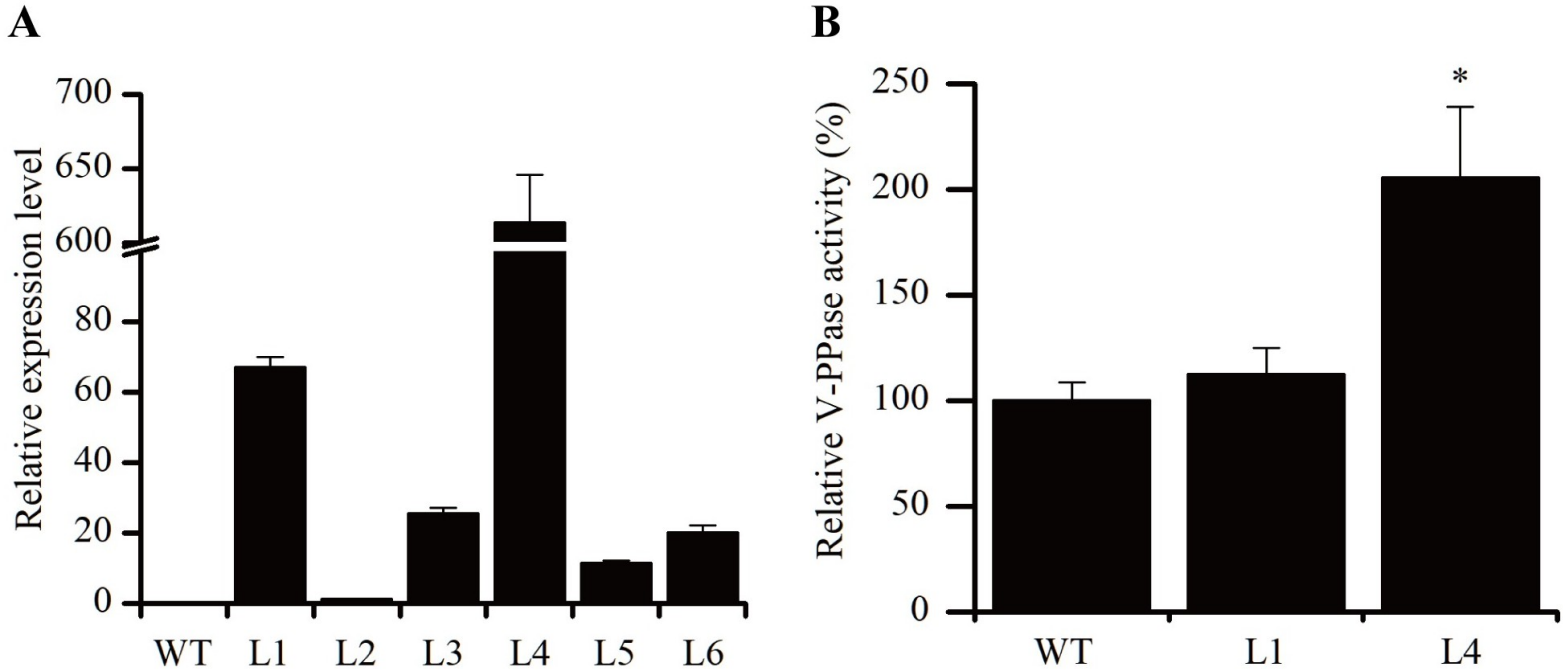
The mRNA transcript levels of *BnVP1* **(A)** and V-PPase activity **(B)** in the roots of the T_3_ generation of transgenic *Arabidopsis* lines. WT: wild type *Arabidopsis*; L1-L6: *BnPCS1* transgenic *Arabidopsis* seedlings. Data are shown as the mean ± SE of three biological replicates. The asterisk represents a statistically significant difference when compared with the wild type (*P*<0.05).

To investigate the role of *BnVP1* in detoxifying Cd stress, we constructed the *BnVP1* overexpression vector pBI121-BnVP1 driven by the CaMV 35S promoter and transformed it into *Arabidopsis* (Col-0). positive transgenic lines were obtained through kanamycin resistance screening and PCR analysis (S3 and S4 Figs). The qRT-PCR results revealed varying expression levels of *BnVP1* among the transgenic lines, designated as L1, L2, L3, L4, L5, and L6. Notably, line L4 exhibited the highest expression level of *BnVP1*, followed by line L1 (Fig 4A). Furthermore, we assessed the V-PPase activity in lines L1 and L4. The results indicated that the V-PPase activity of line L4 was significantly higher than that of the wild type (WT). Although The V-PPase activity in line L1 was slightly higher than that of WT, the difference was not statistically significant (Fig 4B).

### *Arabidopsis* transgenic line L4 showed enhanced Cd tolerance and accumulation

**Fig 5:**
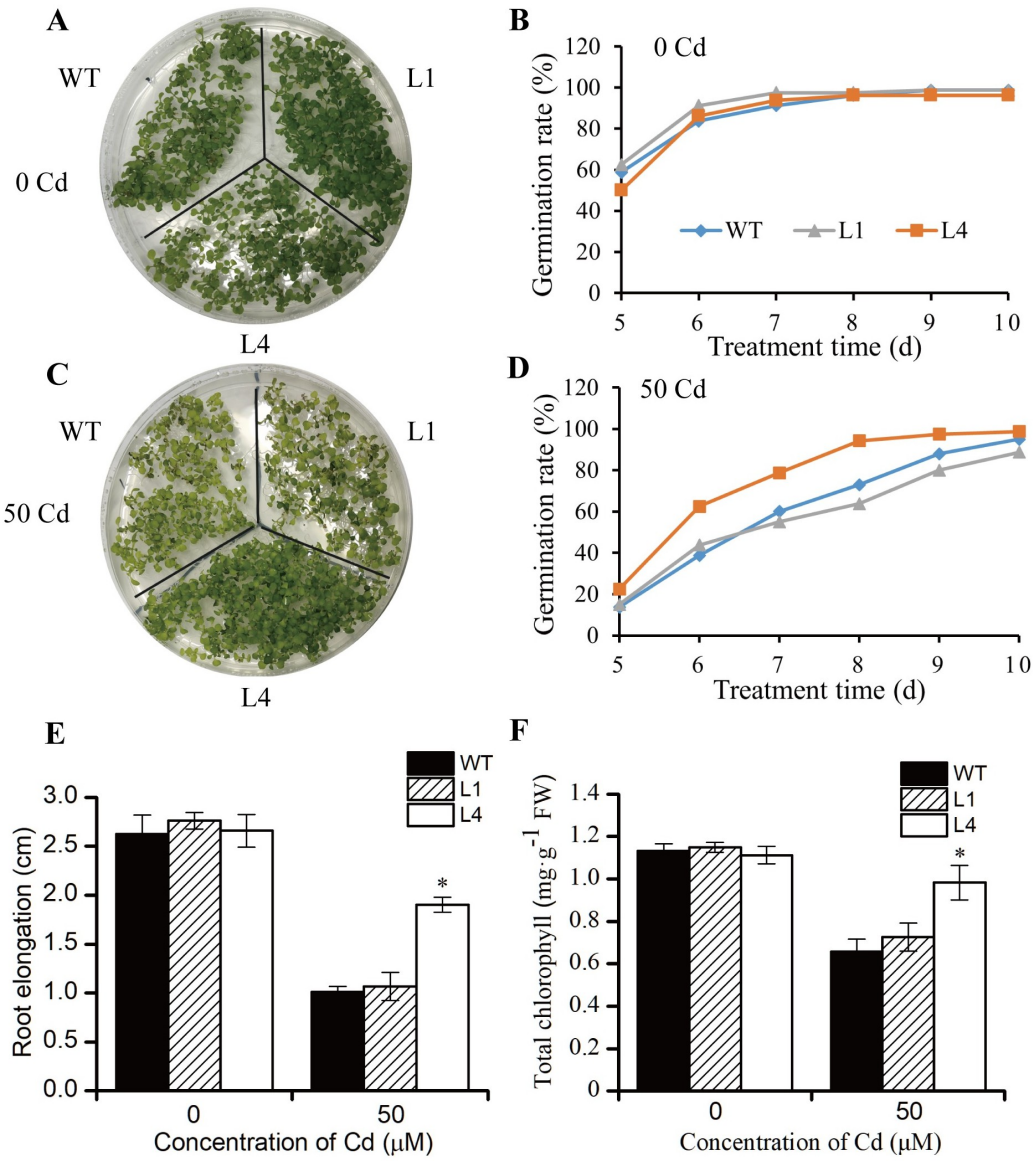
Cd tolerance analysis of the wild type and *BnVP1* transgenic *Arabidopsis* at the seed germination stage. **(A-D)** The phenotypes and germination rates of WT and transgenic *Arabidopsis* lines under 0 or 50 μM Cd treatment at the germination stage. **(E, F)** The root elongation and total chlorophyll content of WT and transgenic *Arabidopsis* lines treated with 0 or 50 μM Cd for 10 days. Data are shown as the mean ± SE of three biological replicates. The asterisk represents a statistically significant difference when compared to the wild type (*P*<0.05).

To analyze the impact of Cd stress on seed germination and growth, we germinated seeds from the T_3_ generation obtained from WT, L1 and L4 on an 1/2 MS medium containing 0 or 50 μM Cd. No obvious differences were found in the germination rates of WT, L1 and L4 in the normal 1/2 MS medium (Fig 5A and 5B). However, when the seeds were grown on an 1/2 MS medium containing 50 μM Cd, the germination speed of WT, L1 and L4 all slowed down, despite the latter showing a faster germination rate likely associated with higher V-PPase activity (Fig 5C and 5D). The root elongation of the WT, L1 and L4 lines showed no differences in growth on the normal l/2 MS medium, but was inhibited when exposed to Cd stress in all three lines. However, the inhibitory effects on the L4 line were significantly less than those of WT, while the root elongation of L1 was slightly higher (but not significantly so) than that of the WT (Fig 5E). Phenotypic observation showed that the leaves of the transgenic line L4 were still green, while those of both WT and L1 turned to a yellowish green color (Fig 5C). To illustrate this phenotype, the total chlorophyll content of *Arabidopsis* seedlings was determined. We found that the total chlorophyll content of WT, L1 and L4 all decreased following Cd exposure, but that the total chlorophyll content of L4 was significantly higher than the WT, while L1’s was slightly higher (but not significantly so) than WT (similar V-PPase activity) (Fig 5F).

### Overexpression of *BnVP1* promotes Cd accumulation and reduces membrane damage under Cd stress

**Fig 6:**
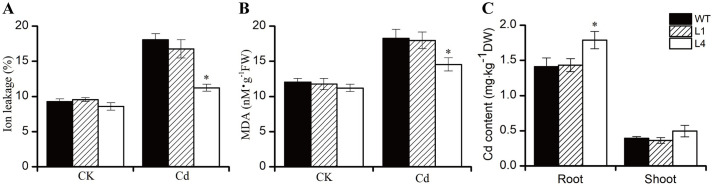
The ion leakage **(A)** and MDA content **(B)** in the roots; and the Cd content **(C)** in the roots and shoots of 4-week-old WT and transgenic *Arabidopsis* seedlings treated with 0 or 50 μM of Cd for one week. Data are shown as the mean ± SE of three biological replicates. The asterisk represents a statistically significant difference when compared with the wild type (*P*<0.05).

Maintaining the integrity of cell membranes under stress conditions is crucial for the mechanisms controlling heavy metal tolerance. Leakage of ions from cell membranes and the accumulation of MDA (malondialdehyde), an indicator of lipid peroxidation, in plant tissues are important measures reflecting the degree of cell membrane damage under Cd exposure. In this study, we assessed the degree of ion leakage rate and MDA content in the roots of WT and two transgenic lines after one week of Cd application (0 or 50 μM). We observed a significant increase in the rate of ion leakage in the roots of all tested lines under Cd exposure. However, the ion leakage rate in line L4 was significantly lower than that in WT, indicating improved membrane integrity. Line L1 exhibited a slightly lower ion leakage rate compared to WT, although the difference was not statistically significant (Fig 6A). The changes in MDA content correlated with the alterations in ion leakage (Fig 6B). To investigate the effects of *BnVP1* on Cd accumulation, we determined the Cd content in roots and shoots of 4-week-old WT and transgenic *Arabidopsis* seedlings treated with 0 or 50 μM Cd. We found that the Cd content in the roots of both transgenic lines was higher compared to WT. Furthermore, the increased Cd content in line L4, which exhibited higher V-PPase activity, reached a significant level, being 1.27-fold higher than in WT. However, overexpression of *BnVP1* did not significantly affect the Cd content in the shoots of both transgenic lines (Fig 6C). These findings suggest that ectopic expression of *BnVP1* reduces the damage caused by Cd to cell membranes, thereby enhancing Cd tolerance in plants. Additionally, it promotes Cd accumulation in the roots of transgenic *Arabidopsis* seedlings, particularly in line L4, which has higher V-PPase activity.

## Discussion

The plant V-PPase, along with vacuolar-ATPase (V-ATPase), plays a central role in vacuole acidification, energizing the active transport of ions across the tonoplast, and coordinately regulating cell turgor pressure and elongation [26]. Since the membrane-bound potassium (K^+^) and magnesium (Mg^2+^) ion-stimulated V-PPase was first isolated from *Beta Vulgaris* by Karlsson [27] in 1975, researchers have subsequently isolated the full-length cDNA of V-PPase genes from *Oryza sativa* [28], *Arabidopsis thaliana* [29], *Nicotiana tabacum* [30], *Vigna radiata* [31], *Thellungiella halophila* [32] and *Hordeum vulgare* [33]. Ramie, also known as China grass, whose fibers serve as important raw material for the textile industry, is widely cultivated in southern China due to its rapid growth, high productivity, adaptability, and strong stress resistance [12, 34]. However, studies on V-PPase genes in ramie have been relatively limited. In this study, the full-length V-PPase gene *BnVP1* was isolated from ramie using the RACE method. Previous research has indicated that the amino acid sequences of plant V-PPase are highly conserved across different plant species, typically containing 15 to 17 transmembrane regions due to their extremely hydrophobic nature [35]. Our results demonstrated that the deduced BnVP1 protein shares at least 90 percent amino acid identity with those from other plant species and comprises 15 transmembrane regions. Furthermore, three highly conserved domains—CS1, CS2, and CS3—associated with the V-PPase family were identified in the BnVP1 protein. The topological model of V-PPase, initially proposed by Maeshima [25], suggests that V-PPase consists of approximately 14 transmembrane regions and three highly conserved domains: CS1, CS2, and CS3. The CS1 domain harbors a conserved sequence (DVGADLVGKVE) found across various plant species, which may serve as the substrate binding site of V-PPase. Additionally, three other conserved sequences (DDPR, VGDN, and GDTIGD) present in CS1 and CS3 domains together constitute the catalytic core. These conserved motifs are crucial for maintaining the stability of V-PPase activity [31]. In BnVP1, the conserved sequences CS1, CS2, and CS3 were all identified, indicating that the BnVP1 protein exhibits the characteristics of the plant V-PPase family and likely performs similar functions to V-PPase genes in other plants.

Recent research suggests that V-PPase proteins can be categorized into two groups, namely Type I V-PPase and Type II V-PPase, based on their localization within plant cells [36]. Type I V-PPase, primarily localized on the tonoplast, is considered the key enzyme involved in PPi hydrolysis and vacuolar acidification, while Type II V-PPase is present in low concentrations and mainly found in the Golgi apparatus of plant cells [37]. In our study, the prediction of protein subcellular localization indicated that BnVP1 is primarily targeted to the tonoplast. Furthermore, phylogenetic tree analysis revealed that BnVP1 shares closer relationships with twelve previously reported type I V-PPase proteins rather than type II V-PPase proteins. Based on these findings, we propose that BnVP1 belongs to the Type I V-PPase family.

The prediction of promoter cis-acting elements of *BnVP1* identified multiple abiotic stress response elements and hormone (Methyl Jasmonate, MeJA) response elements, suggesting that *BnVP1* likely participates in the response of ramie to Cd stress. Kabala et al. [38] reported that the expression of the *CsVP* gene and PPi hydrolysis in cucumber roots was insensitive to Cd exposure but sensitive to Cu. However, in *Hordeum vulgare* and *Arabidopsis thaliana*, Cd treatments significantly induced the expression of V-PPase genes [9, 39]. In our study, gene expression pattern analysis of *BnVP1* in ramie under Cd stress using qRT-PCR revealed that the transcription level of *BnVP1* increased rapidly and reached its maximum within the first 6 hours following Cd exposure, both in the roots and leaves of ramie. Furthermore, we observed that the response of *BnVP1* to Cd stress in ramie roots was significantly greater than that in the shoots. A Previous study has shown that overexpression of *TaVP1* from wheat in tobacco restricted Cd transport from root to shoot and led to Cd accumulation in the roots of transgenic plants [10]. Overexpression of the *OVP1* gene from rice has been shown to increase the Cd content in roots and decrease it in shoots of transgenic rice plants [40]. Our results suggest that *BnVP1* in ramie roots may function similarly in restricting Cd translocation from roots to shoots. A Previous study has demonstrated that MeJA treatments can significantly reduce the ratio of free Cd ions in wheat, thereby restricting Cd translocation from roots to shoots [41]. In our study, the expression of *BnVP1* was significantly induced by MeJA. This may partly explain why MeJA treatment yielded the observed results, as the high expression level of *BnVP1* under MeJA treatment could enhance Cd accumulation in roots while restricting Cd translocation from roots to shoots.

In order to test its potential for Cd tolerance regulation, *BnVP1* was overexpressed in Arabidopsis under the control of the Cauliflower mosaic virus 35S promoter. The transcription level of *BnVP1* gene and V-PPase activity in different transgenic lines (T3 generation) were determined. Although the *BnVP1* gene was highly expressed in both L1 and L4 lines, the V-PPase activity in L1 line was not significantly altered, while the V-PPase activity in L4 line was significantly improved by 2.1-fold compared to WT. Due to the characteristics of *Agrobacterium tumefaciens*-mediated method, the *BnVP1* gene was randomly integrated into the *Arabidopsis* genome. The insertion site effects may induce post-transcriptional gene silencing (PTGS) through DNA methylation or cosuppression, which make the expression or translation of *BnVP1* instability [42, 43]. However, the high level of V-PPase activity in L4 line indicated that *BnVP1* was successfully overexpressed. The following experiments indicated that the L4 line showed improved plant growth and Cd accumulation. We observed that the *BnVP1* transgenic line L4 demonstrated significantly enhanced growth performance in terms of germination rate, root elongation, and total chlorophyll content compared to wild-type plants under Cd exposure (Fig 5). It is well-documented that Cd inhibits chlorophyll biosynthesis, thereby slowing down plant growth [44, 45]. To further investigate this, we measured the total chlorophyll content in WT and *BnVP1* transgenic lines under Cd treatments. The results revealed higher chlorophyll content in the transgenic lines, particularly in L4, compared to WT. We hypothesize that the overexpression of *BnVP1* in *Arabidopsis* plants facilitated the translocation of Cd ions from the cytoplasm into the vacuole, thereby mitigating the adverse effects of Cd on chlorophyll biosynthesis and promoting the production of more photosynthate, ultimately resulting in better germination rates in wild-type plants. However, the differences in growth rate, root elongation, and total chlorophyll content between WT and the L1 transgenic line were not significant. This could be attributed to the lower expression level of *BnVP1* in the L1 line (with a relative expression level of 63), and the slightly higher V-PPase activity in line L1, which did not reach a significant level (Fig 4).

The phytochelatin-dependent pathway has long been recognized as one of the most crucial detoxification mechanisms for Cd, involving ion compartmentation. Genes associated with this pathway have been introduced into plants for phytoremediation purposes [23, 46]. However, mounting evidence suggests that heavy metal detoxification can also occur through phytochelatin-independent tonoplast-localized transporters. These transporters sequester heavy metals into the vacuole via direct proton exchange facilitated by metal/H^+^ antiporters such as cation/H^+^ exchangers (CAXs) and magnesium proton exchangers (MHXs) [47–50]. The transportation of Cd directly into vacuoles by these cation/H^+^ antiporters is energized by the pH gradient established by proton pumps. V-PPase, in conjunction with ATPase, is recognized as the primary enzyme responsible for establishing the pH gradient between the vacuole and cytoplasm. Transgenic tobacco and *Arabidopsis* plants that ectopically express the wheat V-PPase gene have shown improved performance and displayed enhanced Cd and Cu accumulation in both roots and shoots compared to wild-type plants [10, 51]. Overexpression of *OVP1* has been shown to enhance the vacuolar sequestration of Cd and Zn in the roots of transgenic rice and improve rice growth compared to WT under Cd treatment [40]. In our study, we found that the Cd content in the roots of line L4, which exhibits significantly higher V-PPase activity than WT, is 1.27-fold higher than in WT, but not in the shoots. These results suggest that overexpression of *BnVP1* can significantly promote Cd accumulation in roots but not in shoots.

Most plant species are sensitive to Cd, even at low concentrations. Cd stress induces the generation of excessive ROS [52], which compromise the integrity of the cell membrane system and lead to elevated electrolyte leakage through membrane lipid peroxidation. As the primary final products of lipid oxidation by ROS, the levels of malondialdehyde (MDA) and ion leakage are typically used to reflect the degree of oxidative stress in plant cells under various environmental stresses [53]. In this study, the ion leakage rate and MDA content in V-PPase transgenic lines were lower than those in WT, especially in transgenic line L4, which exhibited significantly higher V-PPase activity than WT and the L1 transgenic line. The ion leakage rate and MDA content of line L4 were significantly 37.8% and 20.4% lower than WT, respectively (Fig 6). These results indicate that overexpression of *BnVP1* in *Arabidopsis* alleviated the damage to the membrane system caused by Cd exposure, which can be considered an important factor explaining the better growth performance of the transgenic lines under Cd treatments. It is of interest to explore whether *BnVP1* rescues the Cd-sensitive phenotype of V-PPase gene mutant in the future study.

## Conclusions

In this study, we isolated the *BnVP1* gene, a vacuolar H^+^-pyrophosphatase gene in *Boehmeria nivea*, which contains fifteen predicted transmembrane domains. Sequence analysis indicated that *BnVP1* belongs to the Type I V-PPase family. qRT-PCR assays showed that the expression of *BnVP1* was significantly induced by Cd and MeJA. Heterologous expression of *BnVP1* in transgenic *Arabidopsis* line L4 significantly improved the activity of V-PPase in plants. The growth performance, root elongation, and total chlorophyll content of transgenic line L4 were all improved compared to WT. Overexpression of *BnVP1* alleviated the degree of membrane lipid peroxidation and ion leakage rate, and significantly promoted Cd accumulation in the roots of transgenic *Arabidopsis* seedlings.

## Supporting information

S1 Fig Gel electrophoresis of PCR products of the *BnVP1* gene and its promoter region in ramie. M: Marker; 1: 3’RACE PCR product; 2: ORF PCR product; 3: Promoter PCR product.
(DOCX)

S2 Fig Nucleotide and inferred amino acid sequence of *BnVP1* from *Boehmeria nivea*. The nucleotides are numbered on the left. The inferred amino acid residues are shown under the corresponding codons. The Asterisk indicates the stop codon.
(DOCX)

S3 Fig Kanamycin resistant screening of *BnVP1* transgenic *Arabidopsis thaliana* seedlings. T0 seeds of transgenic *Arabidopsis thaliana* seedlings were germinated on a MS medium supplemented with 30 mg·L^-1^ kanamycin for screening. Transgenic lines remained green, while the WT type turned to a yellow color.
(DOCX)

S4 Fig Identification of transgenic *BnVP1 Arabidopsis thaliana* seedlings by VP1-35S-F/VP1-SP-R primer. M: Trans2K Plus II DNA Marker; P: positive plasmid control; CK: no template negative control; WT: common seedling; L1-L7: *BnVP1* transgenic seedlings. The pBI121-BnVP1 vectors were subjected to *Agrobacterium tumefaciens*-mediated genetic transformation into *Arabidopsis thaliana*. All overexpressing 35S::BnVP1 transgenic lines (T1 generation) were verified by PCR using the VP1-35S-F and VP1-SP-R primers.
(DOCX)

S1 Table The list of primers used in this study. (DOCX)
