# Supplementary material for: BnVP1, a novel vacuolar H+ pyrophosphatase gene from Boehmeria nivea confers cadmium tolerance in transgenic Arabidopsis
Source: PLoS One. 2024 Aug 19;19(8):e0308541. doi: 10.1371/journal.pone.0308541 (PMC11332915; doi:10.1371/journal.pone.0308541)
Supplement: S1 Fig — M: Marker; 1: 3’RACE PCR product; 2: ORF PCR product; 3: Promoter PCR product. (DOCX) [file pone.0308541.s001.docx]

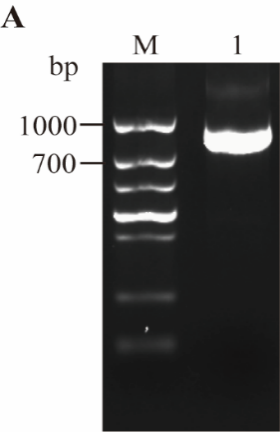

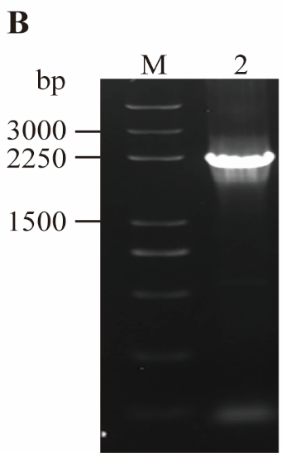

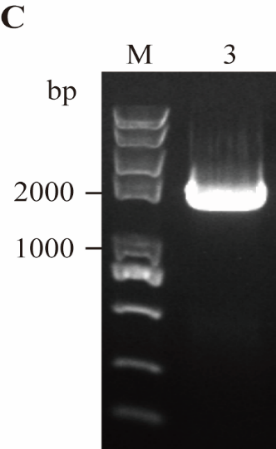


**S1 Fig. Gel electrophoresis of PCR products by PCR amplification of *BnVP1* gene and its promoter in ramie.** M: Marker; 1: 3’RACE PCR product; 2: ORF PCR product; 3: Promoter PCR product
