# Supplementary material for: BnVP1, a novel vacuolar H+ pyrophosphatase gene from Boehmeria nivea confers cadmium tolerance in transgenic Arabidopsis
Source: PLoS One. 2024 Aug 19;19(8):e0308541. doi: 10.1371/journal.pone.0308541 (PMC11332915; doi:10.1371/journal.pone.0308541)
Supplement: S2 Fig — The nucleotides are numbered on the left. The inferred amino acid residues are shown under the corresponding codons. The Asterisk indicates the stop codon. (DOCX) [file pone.0308541.s002.docx]

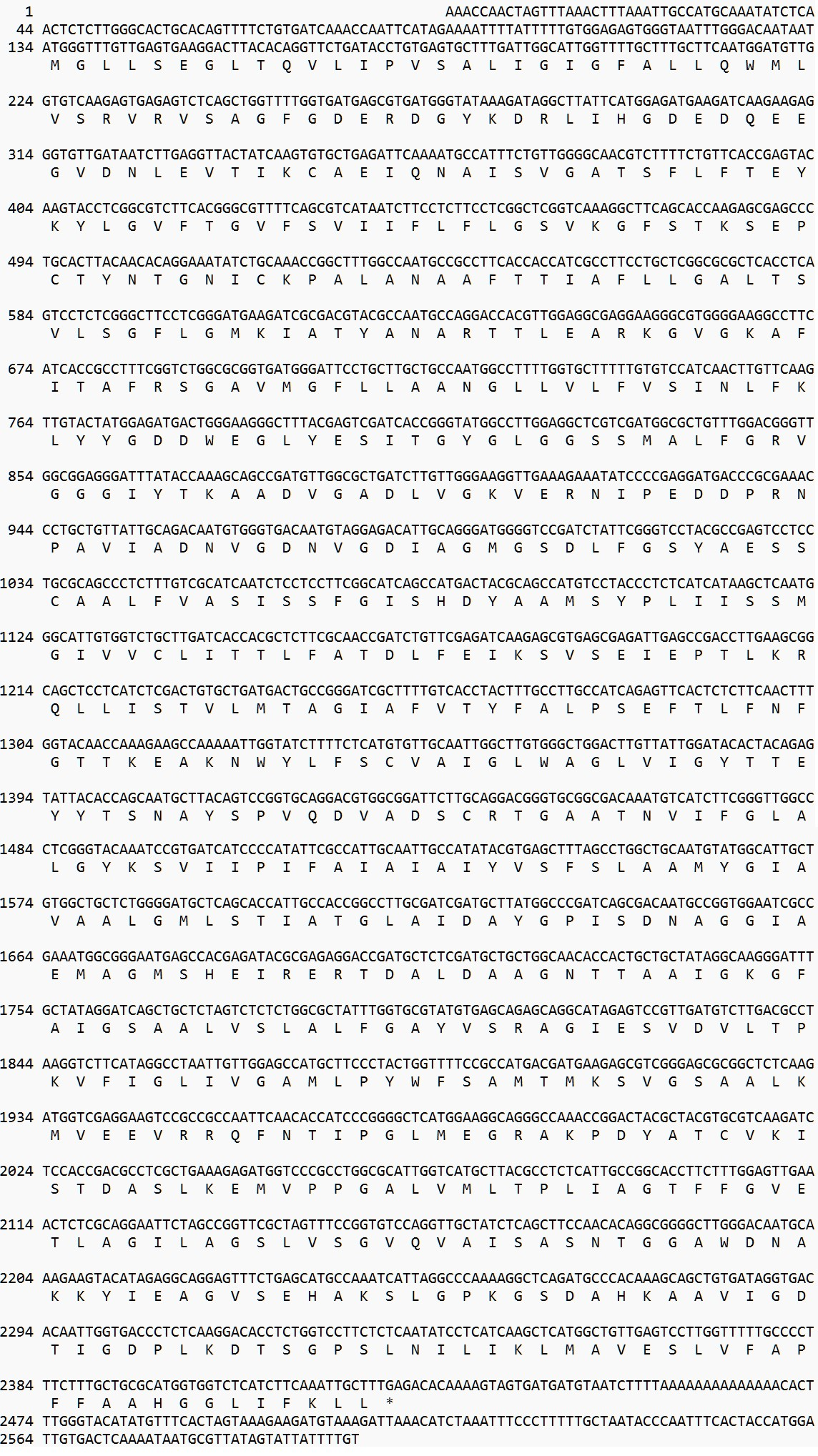


**S2 Fig. Nucleotide and deduced amino acid sequence of *BnVP1* from *Boehmeria nivea.*** Nucleotides were numbered on the left. The deduced amino acid residues were showed under the corresponding codons. Asterisk indicates the stop codon.
