# Supplementary material for: BnVP1, a novel vacuolar H+ pyrophosphatase gene from Boehmeria nivea confers cadmium tolerance in transgenic Arabidopsis
Source: PLoS One. 2024 Aug 19;19(8):e0308541. doi: 10.1371/journal.pone.0308541 (PMC11332915; doi:10.1371/journal.pone.0308541)
Supplement: S3 Fig — T0 seeds of transgenic Arabidopsis thaliana seedlings were germinated on a MS medium supplemented with 30 mg·L-1 kanamycin for screening. Transgenic lines remained green, while the WT type turned to a yellow color. (DOCX) [file pone.0308541.s003.docx]

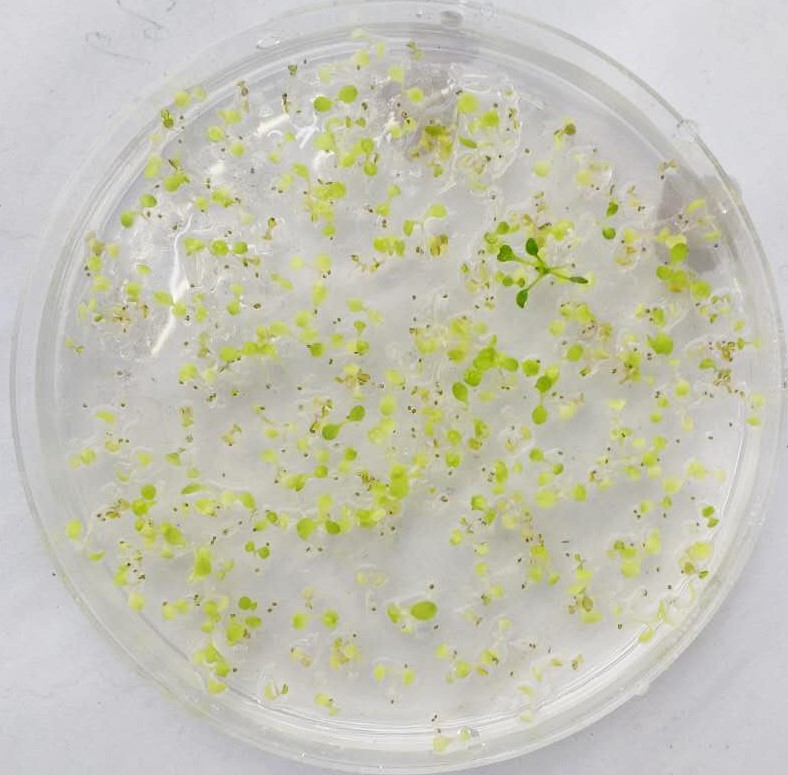

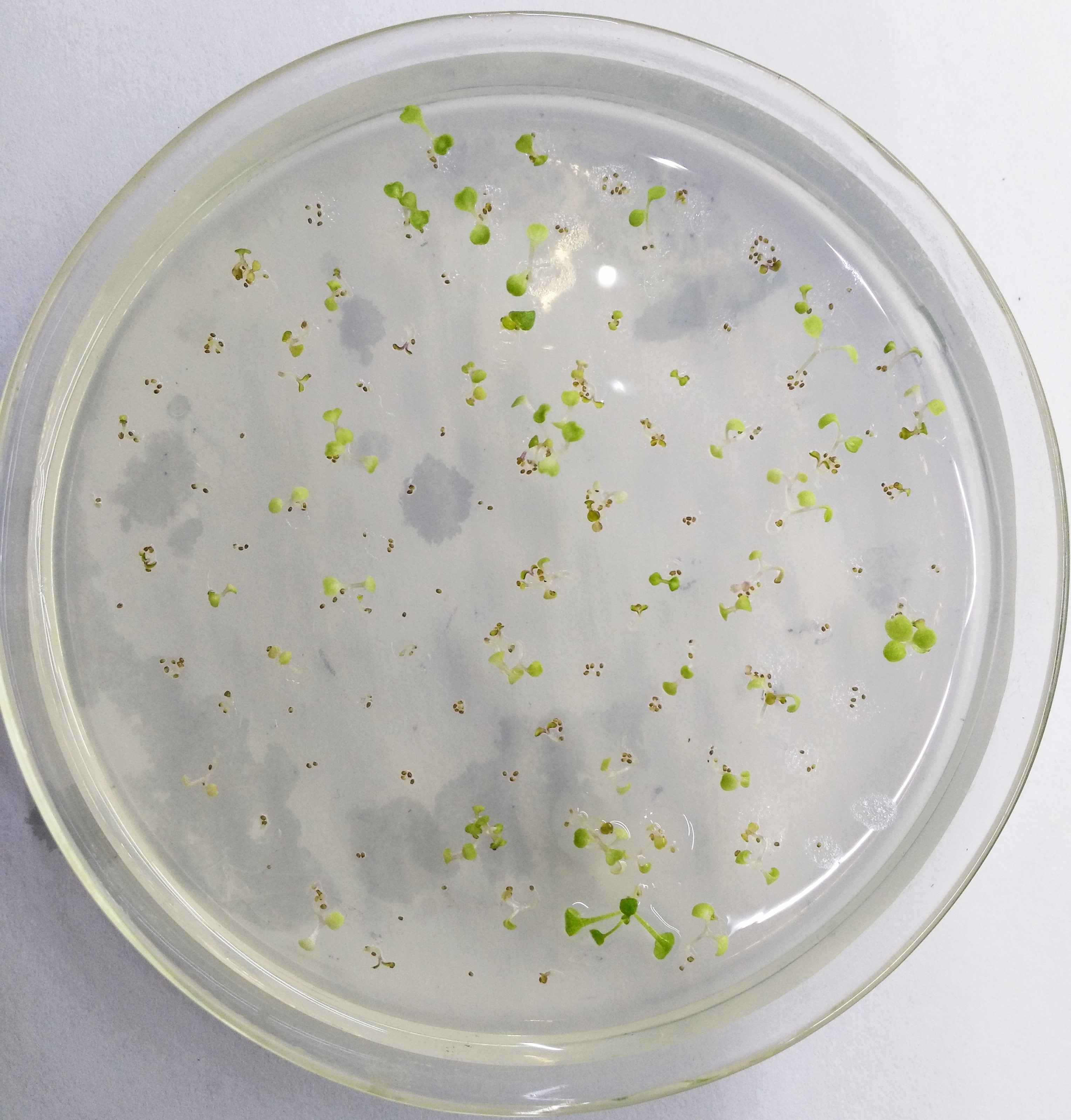


**S3 Fig. Kanamycin resistant screening of *BnVP1* transgenic *Arabidopsis thaliana* seedlings.** T0 seeds of transgenic *Arabidopsis thaliana* seedlings were germinated on MS medium supplemented with 30 mg･L^-1^ kanamycin for screening. Transgenic lines remained green, while the WT type turned yellow.
