# Supplementary material for: BnVP1, a novel vacuolar H+ pyrophosphatase gene from Boehmeria nivea confers cadmium tolerance in transgenic Arabidopsis
Source: PLoS One. 2024 Aug 19;19(8):e0308541. doi: 10.1371/journal.pone.0308541 (PMC11332915; doi:10.1371/journal.pone.0308541)
Supplement: S4 Fig — M: Trans2K Plus II DNA Marker; P: positive plasmid control; CK: no template negative control; WT: common seedling; L1-L7: BnVP1 transgenic seedlings. The pBI121-BnVP1 vectors were subjected to Agrobacterium tumefaciens-mediated genetic transformation into Arabidopsis thaliana. All overexpressing 35S::BnVP1 transgenic lines (T1 generation) were verified by PCR using the VP1-35S-F and VP1-SP-R primers. (DOCX) [file pone.0308541.s004.docx]

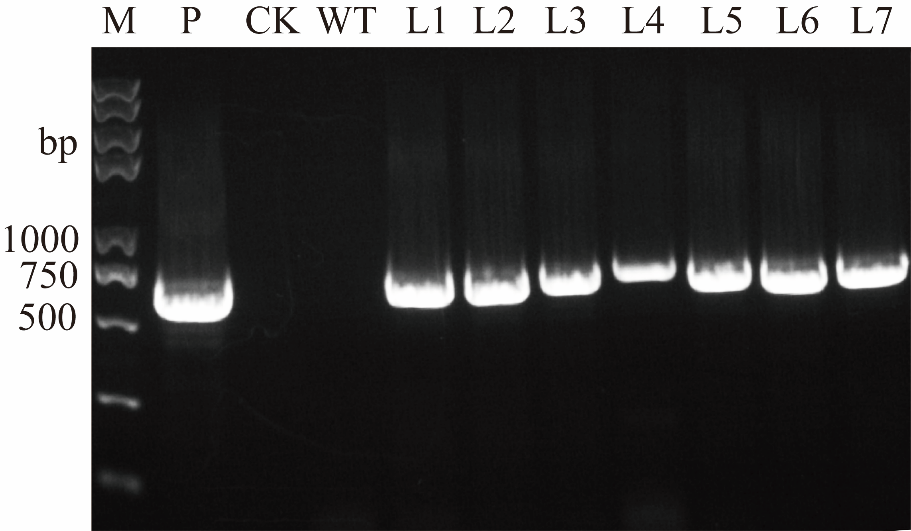


**S4 Fig. Identification of transgenic *BnVP1* transgenic *Arabidopsis thaliana* seedlings by VP1-35S-F/VP1-SP-R primer.** M: Trans2K Plus II DNA Marker; P: positive plasmid control; CK: no template negative control; WT: common seedling; L1-L7: *BnVP1* transgenic seedlings. The pBI121-BnVP1 vectors were transferred by *Agrobacterium tumefaciens*-mediated genetic transformation into *Arabidopsis thaliana*. All overexpressing 35S::BnVP1 transgenic lines (T1 generation) were verified by PCR using VP1-35S-F and VP1-SP-R primers.
