# Supplementary material for: BnVP1, a novel vacuolar H+ pyrophosphatase gene from Boehmeria nivea confers cadmium tolerance in transgenic Arabidopsis
Source: PLoS One. 2024 Aug 19;19(8):e0308541. doi: 10.1371/journal.pone.0308541 (PMC11332915; doi:10.1371/journal.pone.0308541)
Supplement: S1 Table — (DOCX) [file pone.0308541.s005.docx]

**S1 Table. List of primers used in the study**

| **No.** | **Primer name** | **Direction** | **Sequence (5′→3′)** | **Primer purpose** |
| --- | --- | --- | --- | --- |
| 1 | VP1-3FO | Forward | CGGGAATGAGCCACGAGATACG | 3'RACE amplification |
| 2 | VP1-3FI | Forward | AGAGCAGGCATAGAGTCCGTTG |  |
| 3 | VP1-F | Forward | ATGGGTTTGTTGAGTGAAGGACTTACACAGG | Full length verifying |
| 4 | VP1-R | Reverse | TCAAACCTGGACACCGGAAACTAGCGAACCG |  |
| 5 | VP1-PF | Forward | AGTTATCTCTTCTATCTATCTTGAG | Promoter amplification |
| 6 | VP1-PR | Reverse | CCCATCACGCTCATCACC |  |
| 7 | VP1-qF | Forward | GCTTGTGGGCTGGACTTGTTA | Real-time PCR |
| 8 | VP1-qR | Reverse | CACGGATTTGTACCCGAGGG |  |
| 9 | BnActin-F | Forward | GTTGAACCCTAAGGCTAACAGAG | Internal reference primer |
| 10 | BnActin-R | Reverse | GGAATCCAGCACGATACCAG |  |
| 11 | VP1-BamHI-F | Forward | cgGGATCCATGGGTTTGTTGAGTGAAGGACTTACACAGG | Recombinant vector construction |
| 12 | VP1-HindIII-R | Reverse | cccAAGCTTGTGTCTCAAAGCAATTTGAAGATGAGACC |  |
| 13 | VP1-35S-F | Forward | CATTGCCCAGCTATCTGTCACTTT | Positive seedlings screening |
| 14 | VP1-SP-R | Reverse | GCTGAAAACGCCCGTGAAGAC |  |
| 15 | AtActin-F | Forward | GCACCACCTGAAAGGAAGTACA | Internal reference primer |
| 16 | AtActin-R | Reverse | CGATTCCTGGACCTGCCTCATC |  |
